# Queer and feminist reflections on sextech

**DOI:** 10.1080/26410397.2023.2246751

**Published:** 2023-09-15

**Authors:** Kath Albury, Zahra Stardust, Jenny Sundén

**Affiliations:** a Professor, Department of Media and Communication, Swinburne University of Technology, Melbourne, Australia.; b Research Fellow, School of Communication, Queensland University of Technology, Queensland, Australia; c Professor, School of Culture and Education, Södertörn University, Stockholm, Sweden

## Introduction

A recent article in *The Economist* suggested that the rapidly growing global market for sexual pleasure and wellbeing-focused devices and technologies would likely increase even more in 2023,^1^ projected to reach 40 billion US dollars. This market growth has coincided with a proliferation of industry conferences and hackathons worldwide, such as the *Reimagine Sexuality* conference; the *Love and Sex with Robots* conference; the *Sx Tech EU* conference, and the online tech accelerator *SexTech School* (facilitated by marketer and podcaster Bryony Cole), which has run multiple sessions per year since 2018.^2^

Sextech is a broad umbrella term, encompassing biomedical technologies, therapeutic apps and platforms, and pleasure and entertainment-focused technologies – from vibrators to Virtual Reality erotica, machine-learning powered chatbot “companions” (such as *Replika*), and doll-like sex robots. The term itself is widely attributed to Cindy Gallop, founder of the platform *MakeLoveNotPorn.*^3,4^

The category of sextech often intersects with femtech, a field that focuses on “innovation that supports and improves female health by way of software, products, pharmaceuticals, and technology”^5^ (p. 6). While many sextech products and services target audiences via overtly gendered marketing materials, some specifically seek out users who do not wish to associate their sexual pleasure with a specific gender identity or specific anatomical features.^6^

As sextech founder Andrea Barrica has observed, digital technologies and platforms offer the promise of personalised support, pleasure and information service provision, as an accessible (if market-based) alternative to conservative and/or under-funded public health institutions.^3^ Certainly, mobile apps are used globally to support sexual relationships and health, although usage varies widely across settings and contexts, and is influenced by local attitudes to gender and sexuality.^7^

Queer and feminist researchers interested in the intersection of technology, politics and culture are beginning to explore the ways sextech products and marketing materials represent and engage with sexuality, gender, global markets, human rights and data justice. This work builds on concerns raised by global public health and development practitioners and researchers, who argue that the “disruptive” ethos that underpins tech start-up culture is fundamentally incompatible with values such as sustainability and universal access to healthcare.^8^

This short commentary presents a brief overview of recent literature and popular commentary, including suggestions for new research agendas. We do not seek to quantify the prevalence of sextech use, or evaluate its therapeutic utility. Instead, we adopt a queer feminist sociotechnical approach, reflecting on how cultural assumptions around gender, sexuality, health and pleasure guide and inform sextech markets.

While sex robots have been the subject of both scientific interest and feminist critique (e.g.^9,10^) – as well as concerns related to data privacy^11^ – they currently represent the “luxury” end of the global sextech marketplace.^12^ Consequently, our commentary focuses on questions of data and ethics generated by more mundane, affordable technologies, such as menstrual-tracking apps, sex education chatbots and Bluetooth-enabled vibrators. As digital technologies for sex and pleasure are increasingly networked, both bodies and digital devices are opened up to privacy breeches and data leaks. These highlight the importance of placing community needs for sexual rights and data justice at the centre of sextech futures.

## Technologising sex and gender: pleasure, health and access

The feminist and queer history of sex toys is useful for understanding current discussions of sextech, health, pleasure and wellbeing. As Bo Ruberg^13^ demonstrates in their history of sex dolls, even the most “futuristic” and “scientific” sexual technologies are enmeshed in historical sexual cultures, including historical narratives that are repeated so many times that they become part of popular understanding. They show how the repetition of certain histories of sex dolls and other forms of sextech also disguises the stories of sex workers, women, queer people and people of colour, “whose lives have been bound up with the production and popularisation of sexual technologies since long before these technologies came to be seen as the domain of [Anglo-European] male inventors and consumers” (p. 20).^13^

The lines between sexual health technologies, theoretical and biomedical research technologies and technologies focused on sexual pleasure/entertainment have long been blurred.^14^ Narratives of health and wellness have often served as euphemistic “cover stories” in marketing material promoting sex toys and sexual entertainment technologies and media.^15,16^

In tracing the gendered history of sex toys, Lieberman^17^ chronicles the stories of a few noteworthy male pioneers, including Gosnell Duncan who in the early 1970s made silicone dildos for people with disabilities, but which subsequently became popular for anyone wanting to expand their sex life. Lynn Comella^15^ shows how the increasing economic and sexual independence of women helped create a new market as well as a new sexual public, a development in which lesbian and queer women as retailers and educators had important roles.

While sextech continues to be marketed to specific populations (e.g. people with chronic health conditions, and people recovering from gender-affirmation surgery) as a non-medicalised but implicitly therapeutic support for sexual health and pleasure, in spaces combining consumerism, tech design and sex-positive feminism, it is more likely to be represented as simultaneously sexually empowering and a source of self-knowledge, self-care and wellbeing.^18^

As Mishra and Suresh^19^ observe, these sextech marketing approaches are not exclusive to North American and Anglo-European contexts. Sex education-focused sextech and femtech (including menstrual trackers) have also been promoted as a market- (as opposed to state-)based means of enhancing personal empowerment and reproductive health for cisgender heterosexual women and LGBTQ+ people in the Global South (see also^20^).

Human rights and development-focused agencies (including UNESCO) have explored sextech as a means of redressing deficits in comprehensive sexuality education and on-the-ground health promotion.^21^ Similarly, sexual health organisations in jurisdictions that are subject to significant repression of sexual and reproductive health information have developed Artificial Intelligence-powered sextech (for example Planned Parenthood’s chatbot *Roo*) in an attempt to overcome the legal and geographical barriers that young people face when seeking support.^22^

While technology promises to circumvent a range of barriers to institutionalised education and health service provision thrown up by state and religious regulation of sexual health services, we note that it is not a panacea. Where state-funded schools and hospitals are obliged to provide basic education and health services in the public interest, tech start-up culture is based on assumptions of continuous growth in market share, in order to provide ongoing returns in capital investment.^23^

These conflicting expectations may be resolved via compromise, but may also be fundamentally incompatible. Indeed, a mass embrace of what Morozov^24^ terms “technological solutionism” in sexual and reproductive health may introduce new problems, without fully resolving existing challenges.

## Sextech as a site of (gendered) entrepreneurial aspiration and stigma

Sextech occupies a unique space within global start-up culture.^25^ Like other fields of health tech, it attracts entrepreneurs with medical and/or public health and development backgrounds, who are attracted to the promise of untethering healthcare and wellbeing information and services from bureaucratic institution. It also offers opportunities for members of stigmatised or marginalised sexual communities to translate their lived experience and professional expertise (for example, in the domain of sex work) to share their skills and knowledge with broader communities, and develop new income-generating opportunities.

The high-profile leaders of sextech are women and gender-diverse people. However, opportunities are constrained by the “chokepoints”^26^ built into both global digital commerce platforms, and discriminatory interpretations of “community standards” relating to the marketing of products related to sexuality and reproductive health.^27^ In response to these platform-based restrictions, *TickleLife* founder Shakun Sethi recently launched a suite of alternative digital services, including *Tickle-Charge* – an adult payment gateway for “high risk” merchants – as a means of providing stable and reliable avenues for sextech marketing and distribution.^28^

The impacts of sex-negative responses to sextech may be experienced in quite different ways by diverse stakeholders, including people of colour, gender-diverse people and sex workers.^29^ To date, there has been little scholarly research into the relationship between sexual stigma and the market opportunities and constraints specifically facing founders and developers in the fields of sextech. However, emergent research investigating discrimination against digital sexual information and entertainment content producers offers promising directions for future scholarship in this space.^30,31^

## Data practices in sextech

While not all sextech is digitally-enabled, there is a proliferation of apps and tracking platforms that seek to quantify sexual experience – which raises important concerns regarding data privacy and security.^32^ Additionally, as Burgess et al.^33^ observe, datafied sextoys have been marketed in ways that offer insights into contemporary prevailing narratives relating to gender and sexual pleasure. For example, both the Lioness vibrator and the Lelo F1 masturbation sleeve contain sensors that track genital movements and sensations, which are then visualised for the user via a mobile app interface. But their marketing material describes the purpose of this data visualisation quite differently.

The Lioness – primarily marketed to cisgender women – promises to enhance self-knowledge and intimate connection via data – and also offers users an opportunity to “donate” their data to sexological researchers. As Saunders^34^ argues, these connections to both self-care and quantification connect in interesting ways to post-feminist discussions where both a form of objectivity and distance are key to articulations of female sexuality. In contrast, the Lelo F1 penile masturbation sleeve invites cisgender men to respond to data insights by re-programming their device, enhancing their *own* pleasure.^33^

While not every sextech device collects user data, those that do may collect significant amounts of information. Data is volunteered by users through in-app diaries, detected via sensors employed in smart sex toys or facilitated by vague privacy policies.^35,36^ Where smart vibrators and other sex toys are used in the context of sexual entertainment, they offer new opportunities for what might be termed “collective intimacy”, for example where Bluetooth connection and automation heightens shared experiences for online sexual entertainment audiences.^37^ However, multiple security vulnerabilities have been identified in smart sex toys and user data leaked to advertisers.^38,39^

The development of algorithmic sexual profiles that predict user preferences and behaviour may also have serious implications – including amplifying systemic discrimination in access to education, healthcare, credit and social services.^40^ While an increasing body of literature exists about the racial and gendered impacts of algorithmic profiling,^41,42^ the study of intimate data and sexual profiling is still emerging. The relationship between global platforms, data markets and state-level reproductive and sexual surveillance is a pressing concern.^43^ The creation of databases recording tech users’ sexual preferences, sexual health and sexual behaviour has legal implications, particularly where sexual and reproductive choices are subject to criminal sanctions.^44^

Networked sex toys, such as Bluetooth vibrators, thus actualise the question of what consent means with respect to both sex and data. Within the European General Data Protection Regulation (GDPR), the question of data protection and data privacy takes central stage in ways that have consequences on a global level as data continuously cross borders. To ensure that the standard of explicit consent is met, companies selling networked sex toys need to be clear about what data they will be processing, and for what purpose.^39^

Safe sex within this framework thus becomes a question of keeping one’s *data* safe.^32^ Where this responsibility is pushed onto users (who *must* agree to existing Terms and Conditions in order to log onto devices or platforms), it becomes clear that sextech is a contested site of data justice. Future formative and summative research in the space of sextech for sexual and reproductive health attend both to sextech users’ rights as “data subjects”, and sextech developers’ and founders’ responsibilities for ethical data stewardship.^25^

## Sextech futures

As sex and data are increasingly entangled, our commentary places sexual rights and data justice at the very heart of more hopeful sextech futures. Social change requires a capacity to imagine the world ordered differently, as “no political revolution is possible without a radical shift in one’s notion of the possible and the real” (p. xxiii).^45^

At the same time, as Orrell^8^ puts it, “we need to apply new tech in ways that *minimize* disruption to fledgling and fragile, or underfunded and understaffed institutions and contexts rather than maximize it, as the private sector does” (original emphasis). Such an approach will also require tech developers and marketers to collaborate with and support activists and advocates who are currently confronting sexual stigma, and pushing back against the criminalisation of sex and gender diversity, and reproductive freedoms.

We see precedents for the development of new sexual technologies in the work of disability activists who continue to pioneer future relationships between accessibility and technology.^46^ Critical race scholars have explored how carceral technologies can be resisted and reworked for liberatory purposes.^41^ Marginalised communities have collaboratively produced design principles for more just futures.^47^ Pleasure has been an issue of central political importance for a wide range of political movements and is equally vital in imagining technological futures differently. Pleasure can even be understood as *essential* to social justice, as pleasure, as adrienne maree brown puts it, “is a measure of freedom”.^48^
